# Comparative Morphology of Dendritic Arbors in Populations of Purkinje Cells in Mouse Sulcus and Apex

**DOI:** 10.1155/2013/948587

**Published:** 2013-11-07

**Authors:** Hermina Nedelescu, Mohamed Abdelhack

**Affiliations:** ^1^Okinawa Institute of Science and Technology Graduate University, Okinawa 904-0495, Japan; ^2^University of Antwerp, 2000 Antwerp, Belgium; ^3^Brain Mechanism for Behavior Unit, 1919-1 Tancha, Onna-son, Kunigami-gun, Okinawa 904-0495, Japan

## Abstract

Foliation divides the mammalian cerebellum into structurally distinct subdivisions, including the concave sulcus and the convex apex. Purkinje cell (PC) dendritic morphology varies between subdivisions and changes significantly ontogenetically. Since dendritic morphology both enables and limits sensory-motor circuit function, it is important to understand how neuronal architectures differ between brain regions. This study employed quantitative confocal microcopy to reconstruct dendritic arbors of cerebellar PCs expressing green fluorescent protein and compared arbor morphology between PCs of sulcus and apex in young and old mice. Arbors were digitized from high *z*-resolution (0.25 *µ*m) image stacks using an adaptation of Neurolucida's (MBF Bioscience) continuous contour tracing tool, designed for drawing neuronal somata. Reconstructed morphologies reveal that dendritic arbors of sulcus and apex exhibit profound differences. In sulcus, 72% of the young PC population possesses two primary dendrites, whereas in apex, only 28% do. Spatial constraints in the young sulcus cause significantly more dendritic arbor overlap than in young apex, a distinction that disappears in adulthood. However, adult sulcus PC arbors develop a greater number of branch crossings. These results suggest developmental neuronal plasticity that enables cerebellar PCs to attain correct functional adult architecture under different spatial constraints.

## 1. Introduction

The cerebellum is considered to have a regular arrangement of neuronal cell types produced by the geometric organization of their somata and dendritic trees. In terms of their dendritic arbors, Purkinje cells (PCs) are the most prominent cell type in the cerebellar cortex. Obersteiner [1] reported that these display consistent orientation of dendritic branching with respect to the transverse direction of cerebellar folia [2]. Parallel fibers, which are one of the main sources of input to PCs, were described by Santiago Ramon y Cajal as lying perpendicular to PC dendritic arborizations. In addition to its regularity, the cerebellum is also highly foliated, giving rise to three distinct gross anatomical subdivisions: sulcus, apex, and bank [2–5]. In the sagittal plane, these subdivisions are easily distinguished, since the folia are divided by fissures of varying lengths, creating valleys and summits, separated by less convoluted regions.

Previous work on differences between these subdivisions has elucidated several important features. PC density in the apex is greater than in the sulcus [2, 3]. Studies of sparsely labeled Golgi preparations reported differences in PC branching structure according to their anatomical location. PCs in the sulcus often branch from the somata at an angle of nearly 180°, whereas those in the apex branch tens of microns into the molecular layer [3]. A climbing fiber innervation study demonstrated different connectivity patterns to PCs in the sulcus, indicative of a specialized functional role [4]. More recently, Sudarov and Joyner showed that mouse cerebellar foliation is initiated by formation of “anchoring centers,” created by an inward accumulation of granule cell precursors (gcps) and giving rise to subdivision of the sulcus [5]. Finally, at the single cell level, adult sulcus PCs were shown to retain multiplanar dendritic arbor arrangements [6].

Investigations of morphological, and to a lesser extent, functional relationships of dendritic arbors between cerebellar subdivisions have necessarily focused on single cells. However, determining spatial parameters of neighboring dendritic arbors is important for understanding functional connectivity, particularly of afferent parallel fiber input, across individual PCs residing in different cerebellar subdivisions. Population-level morphological studies have been difficult to execute for several reasons. Only within the last decade has it become possible to label all neighboring neurons of specific cell type with a fluorescent label strong enough to withstand high resolution confocal imaging. Faster scanning speeds, enabling more efficient acquisition times, have been developed only within the last five years. Lastly, classical methods for tracing dendritic morphology are so time-consuming that it is impossible to reconstruct enough cells for appropriate analyses of dendritic morphologies across anatomical regions of interest. Newer methods are being developed [7–9], although few explore the local architecture of neuronal populations [10, 11] and their connectivity to different cell types [12, 13].

This study investigated morphological relationships of dendritic arbors of neighboring PCs in the two most distinct cerebellar regions, sulcus and apex. Unfortunately, to date, no automated algorithm to reliably reconstruct neighboring dendritic arbors has become available. However, with Neurolucida software [14], devised for reconstructing neurons, we adapted a tool that is normally used to draw cell bodies. This was used to efficiently trace dendritic trees labeled with green fluorescent protein (GFP) of *L7-tau-GFP* mice [15] under control of the L7 promoter [16]. The adapted contour protocol allowed us to capture full PC dendritic geometries in one-fourth the time that would have been required with classical tracing methods. These reconstructions permitted quantitative comparative analyses of small populations of neighboring dendritic arbors that hitherto have been impossible.

We show that distinctly different dendritic tree geometries in the sulcus produce greater arbor field overlap among neighboring PCs than in the apex. These findings shed light on functional connectivity of PC dendritic arbor afferent input, relationships between cerebellar cortex subdivisions, and the relationship between developmental plasticity and attainment of adult cerebellar architecture.

## 2. Materials and Methods

### 2.1. Animals

L7-tau-GFP mice [15] were euthanized in the laboratory of Michael Häusser, University College London (UCL). At postnatal day 12 (P 12) (young) and P 150 (old), mice were deeply anesthetized using a cocktail of ketamine/xylazine (80/5 mg/kg, *i.p.*). Transcardial perfusion employed a syringe pump at a perfusion rate of 12 mL/min. Perfusion solution contained 60 mL saline-containing heparin (10 U/mL) and 40–60 mL 4% paraformaldehyde in 0.1 M phosphate buffer (PB) (pH 7.4). This procedure complied with the protocol approved by the UCL Animal Care Committee and with animal care guidelines of the UK Home Office.

Following this perfusion, brains were removed, postfixed overnight with the same fixative, and transferred to a 0.01 M PB solution containing 0.9% sodium chloride (PBS, pH 7.4). Brains were stored in PBS in order to avoid overfixation and possible tissue shrinkage during shipment. Fixed brains were then shipped to the Okinawa Institute of Science and Technology Graduate University (OIST), where they were prepared for confocal microcopy in the laboratories of Bernd Kuhn and Jeff Wickens.

### 2.2. Tissue Preparation and Confocal Microscopy

**Figure 1:**
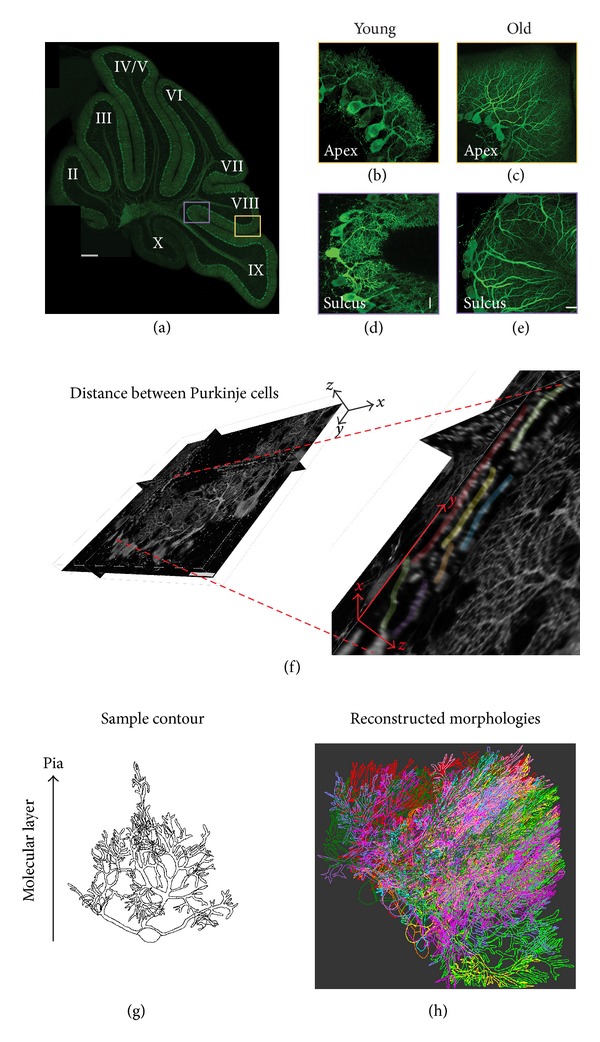
Anatomical sampling, confocal microscopic imaging, and morphological reconstruction of PC dendritic arbors expressing green fluorescent protein. (a) Sulcus lobules VIII-IX and apex lobule VIII were systematically imaged from cerebellar sagittal slices near the midline (vermis). Scale bar: 200 *μ*m. (b) Young mouse (12-day) PC dendritic arbors from apex and (d) sulcus. (c) Old mouse (150-day) PC arbors from apex and (e) sulcus (Scale bars: 20 *μ*m). (f) Example of confocal image demonstrating the distance between neighboring PC dendrites (colored dendrites viewed from the side in zoomed image) as a result of image acquisition with a 0.25 *μ*m step size in the *z*-direction (*z*: image stack depth; *y*/*x*: dendritic tree height and width in the sagittal slice, resp.) (g) Example of dendritic arbor tracing using the adapted Neurolucida (MBF Bioscience) contour function. The extracted geometry is a set of coordinates in 3D space, which can also be applied to nonplanar dendritic structures. (h) Example of neighboring PC dendritic arbor geometries populating the apex. PC somata are 13–20 *μ*m.

At OIST, brains were sliced into 80 *μ*m sagittal sections using a Leica vibratome. Sections containing the cerebellar vermis were collected in cold 0.01 M PBS, mounted onto glass slides, air dried, and cover-slipped using antifade Prolong (Invitrogen) mounting media. To collect samples in a random and systematic manner, only cerebellar sections near the midline were imaged. PC dendritic arbors from the same locations in apex regions of lobule VIII and sulcus regions of lobules VIII and IX were imaged from both young and old cerebella (Figures 1(a)–1(e)).

Cerebellar sagittal sections were imaged with an LSM 710 Carl Zeiss confocal microscope equipped with a 63X (NA 1.46, OIL) objective lens and Argon (458 nm, 488 nm, and 514 nm) lasers. Image acquisition parameters were as follows: an *x*, *y*-size of 0.22 *μ*m/pixel (dimensions *x*: 224.7 *μ*m, *y*: 224.7 *μ*m; image size 1024 × 1024) and a *z*-step size of 0.25 *μ*m. High-resolution confocal image acquisition enabled us to unambiguously resolve dendrites of neighboring PCs in the parasagittal plane, or through the depth of the sagittal slice (Figure 1(f)). Digitized image stacks were acquired with ZEN imaging software (Zeiss).

### 2.3. Digital Extraction of Dendritic Morphologies

Utilizing the continuous contour tracing function of Neurolucida (MBF Bioscience), a surface contour was traced around the outer edge of each PC dendritic tree using a Wacom Cintiq video tablet and digital pen. The video tablet permitted visualization of the confocal stack that contained dendrites of interest. Neurolucida's continuous contour tracing function does not require discontinuous insertion of points but adds points automatically as the experimenter drags the pen along the edge of the dendritic membrane, comparable to drawing on a piece of paper. Digitally, an enclosed hollow contour that accurately delineates the geometry and spatial boundaries of the dendritic arbor was obtained (Figure 1(g)). Somata were drawn in a similar manner and digitally colored to match dendritic contours. These steps were repeated for each dendritic tree and soma in the sulcus and apex of the folia to develop a reconstructed population of neighboring dendrites and somata (Figure 1(h)). All traced PC trees were edited to insure that contours were traced tightly around dendritic trees for the sake of consistency. This contour tracing technique is considerably more efficient than classical reconstruction methods that require the experimenter to adjust the diameter of the cursor at each step and to insert a bifurcation node at each branch point on the dendritic tree.

### 2.4. Experimental Design

Because approximately 10 PCs are found in the sulcus of an 80 *μ*m sagittal cerebellar slice, 10 ± 2 PCs were traced from both sulcus and apex of four mice, for a total of 88 PC dendritic trees. The number of PCs traced per sample was determined by tracing a reference PC in the middle of the sulcus and then reconstructing neighboring PCs within that sulcus subdivision. PCs with dendrites cut off by the confocal image stack (224 *μ*m by 224 *μ*m) were excluded. Since morphological differences between dendritic trees of sulcus and apex are strikingly different, approximately 20 PCs from each age group and anatomical subdivision were sufficient statistically.

### 2.5. Statistical Analyses

 Two important requirements for using Student's *t*-test to compare group means are that samples have normal distributions and equal sizes. Since the number of PCs in each experimental condition was almost equal (10 ± 2 PCs), Student's *t*-test was used to compare means between sulcus and apex PCs for both young and old developmental groups. In cases involving crossed dendritic branches from the same PC, the nonparametric Mann-Whitney *U* test was used because samples did not possess normal distributions and not all PC arbors displayed crossings, rendering sample sizes (number of PCs per group) unequal.

### 2.6. Methodological Considerations

In order to unambiguously resolve distances between dendrites of neighboring cells, high-resolution confocal imaging in the *z*-dimension (depth of the sagittal slice) is critically important. Neighboring PC dendrites are on average 2–4 *μ*m apart in the mediolateral plane [17]. This relatively large separation is because Bergmann glia, basket cells, and other interneurons need to be accommodated as well. In addition, PC dendritic diameters average 0.5–2.2 *μ*m for spiny distal dendrites and 3.45 *μ*m for smooth proximal dendrites [11]. Given these dimensions, with fine optical sectioning, it is fairly simple to resolve the dendrites of neighboring PCs. A recent study reported that PC dendrites touch each other; however, in that study, optical sectioning in the *z*-dimension of the sagittal slice (mediolateral plane) was set to 1 *μ*m, so that PC dendrites appeared to be “sticking to each other” [18].

Confocal acquisition with a small *z*-step size can take time, leading to tissue bleaching if fluorophore expression is not stable and intense. In this study, using a fast laser scanning confocal microscope (Zeiss LSM 710) and L7-tau-GFP transgenic mice that strongly express GFP, we were able to unambiguously resolve dendrites of neighboring PCs without bleaching. Image stack acquisitions took approximately 20 min.

### 2.7. Automated Analysis

**Figure 2:**
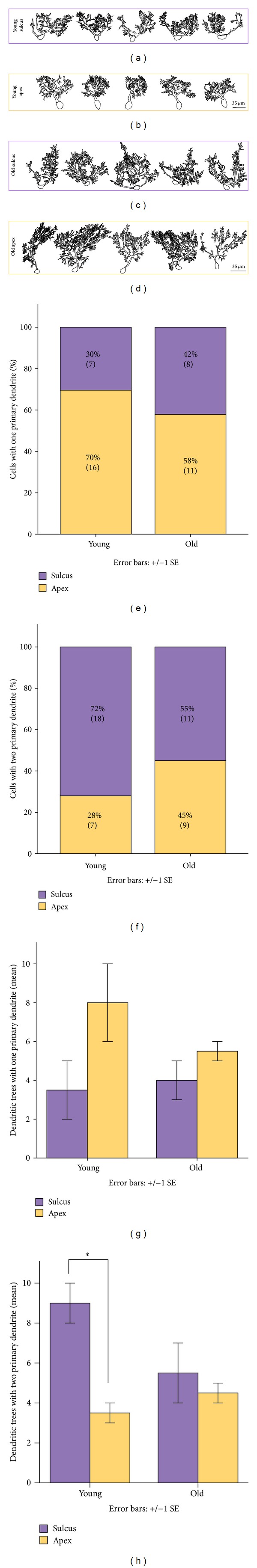
Anatomical location dictates dendritic morphology. (a) Representative morphologies of PC dendritic arbors in the 12-day sulcus and (b) apex, and (c) 150-day sulcus and (d) apex. (e) Percentage of dendritic trees from sulcus and apex having one or (f) two primary dendrites. Note the high frequency of PCs with two primary dendrites in the young sulcus. (g) Mean number of PC arbors with one and (h) two primary dendrites in developing sulcus and apex.

**Figure 3:**
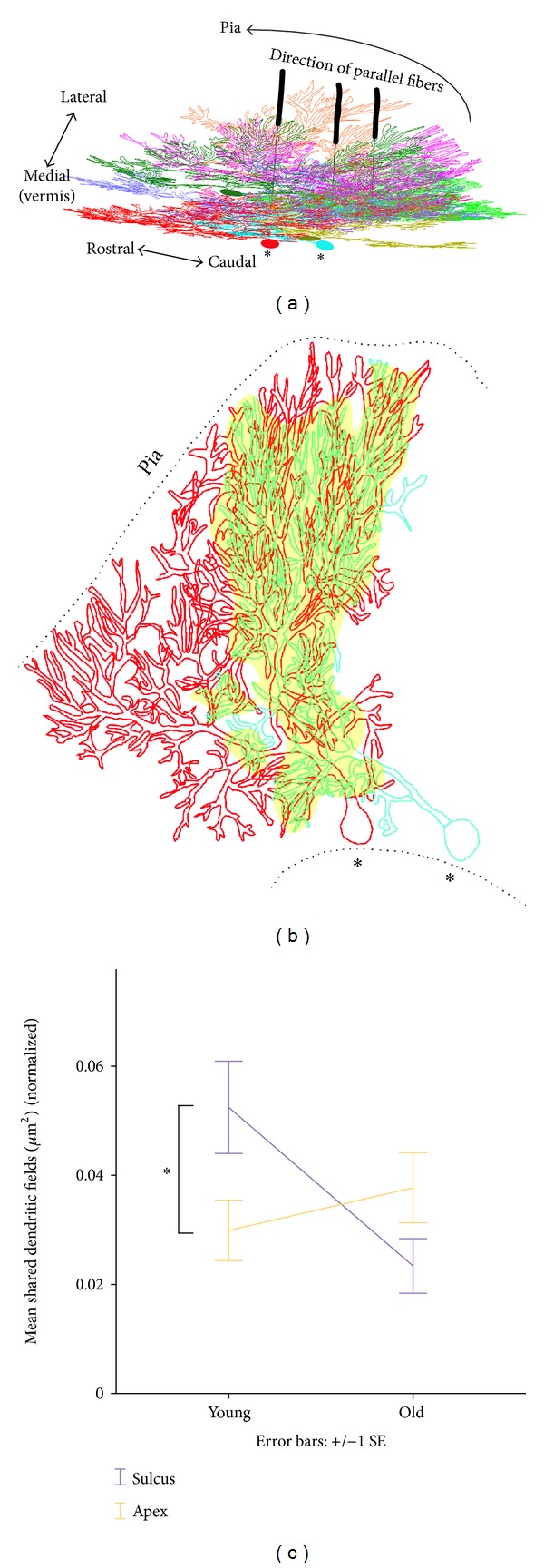
Shared dendritic fields among neighboring PC arbors, illustrating parallel fiber afferent input. (a) Neighboring PC dendritic arbors viewed from the pial surface but tilted to better visualize the architectural organization in the mediolateral plane. (b) Sagittal view of shared dendritic fields between neighboring PC arbors, highlighted in yellow in Panel (a), and most likely the site of shared parallel fiber input. (c) Summary plot of shared dendritic field area normalized by the average dendritic contour area of each PC pair, demonstrating that the effect of age on the amount of shared dendritic field depends on the anatomical location. Normalization eliminated the effect of variable arbor size (young versus old arbors) as a confounding factor *P < 0.05 (*n* = 85 PCs).

**Figure 4:**
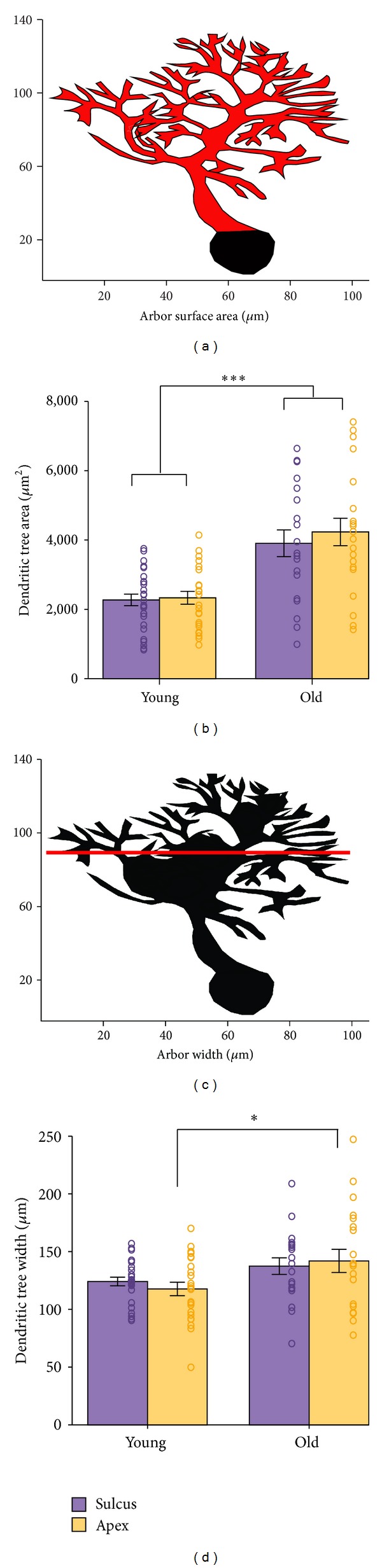
Dendritic arbor morphology in relation to cerebellar subdivision and mouse age. (a) Visualization of the contoured arbor adapted to trace the dendritic tree showing the computed dendritic tree surface area (red). (b) PC dendritic arbor area. Sulcus and apex areas did not differ significantly in either age group; however, young and old arbors were significantly different, ***P < 0.001. (c) Measurement of PC arbor width (red line). (d) PC arbor width. There was a weak tendency for sulcus arbors to be narrower than those of apex, but the difference was not statistically significant; however, old mouse arbors were significantly wider than those of young animals, *P < 0.05 (*n* = 85 PCs). Note the overall heterogeneity.

**Figure 5:**
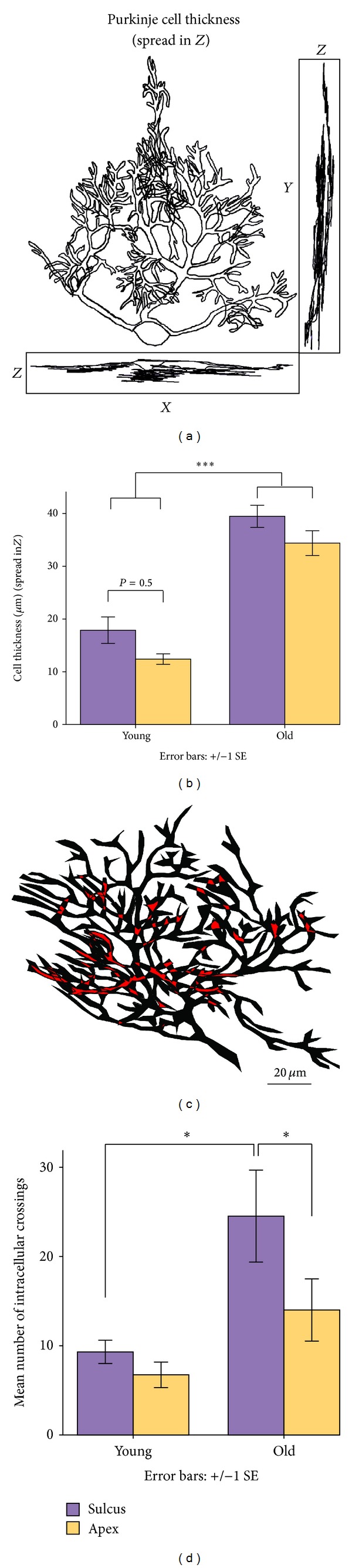
PC thickness and intraarbor dendritic crossings. (a) Morphological reconstruction showing the *XZ*, *YZ*, and *XY* planes to visualize planarity of PC arbors. (b) PC dendritic thickness in the *z*-dimension. There is a tendency for sulcus PC dendrites to achieve a larger spread in the mediolateral plane (*z*-dimension). (c) Graphical representation of a PC dendritic arbor with branches overpassing each other (locations indicated in red, somata excluded). (d) Mean number of overpassing branches for PC arbors in sulcus and apex of young and old mice. There is a significant difference in the mean number of intraarbor crossings in young versus old sulcus, *P* < 0.05, Mann-Whitney *U* test. There is also a significant difference in old sulcus arbors relative to apex (*P* < 0.05, Mann-Whitney *U* test).

 Traced PC dendritic contours containing geometric information in the form of *x*, *y*, *z* coordinates were analyzed with the Polygon Clipper function (Sebastian Hölz, MATLAB Central File Exchange) and with TREES 1.15 (MATLAB toolbox) [9]. In addition, we wrote custom code to extract information about the number of primary and secondary dendrites of each ramified arbor (Figures 2(e)–2(h)), the dendritic field overlap of neighboring arbors (Figures 3(b) and 3(c)), the surface area encompassed by the traced contour of each dendritic arbor (Figure 4(a)), and intracellular dendritic branch crossings (Figures 5(c) and 5(d)).

Shared dendritic field is the overlap area of two neighboring dendritic field arbors (Figure 3(b)). These areas were computed by first triangulating dendritic arbors in 3D to form tetrahedrons. Then, tetrahedrons representing dendritic arbors were clustered, based on their depth within the tissue section. This step facilitated pairing of nearby tetrahedrons (PC dendritic arbors) and avoided accidentally overlapping PC arbors that were actually well separated in the *z*-dimension. Next, overlapping areas of neighboring PC dendritic arbors were computed within each designated cluster. To eliminate variable arbor size as a confounding factor, areas of dendritic field overlap were normalized to the average total dendritic contour of each PC pair ((area of cell 1 + area of cell 2)/2).

Intracellular dendritic branch crossings were computed by detecting sites where branches within the same dendritic tree intersected within a 2D projection. This counted the number of times branches crossed each other within the same arbor.

## 3. Results and Discussion

### 3.1. Comparative Anatomical Differences in PC Morphology Across Cerebellar Subdivisions

Anatomical, physiological and gene expression studies have reported that cerebellar subdivisions function as partitioned modules [19–24]. Various studies have demonstrated gene expression boundaries that subdivide the cerebellar cortex into four transverse zones: anterior, central (CZa and CZb subdivisions), posterior, and nodular, as well as additional topographic units such as the PC stripy bands or microzones [23–28].

A well-established anatomical landmark for describing cerebellar sagittal sections is the lobules (lobules I-X), which are separated by fissures and folds that give rise to sulcus and apex (Figure 1(a)). While the sulcus and apex are prominent topographical subdivisions, their PC dendritic cytoarchitecture and functional diversity have received little attention [3, 6]. Previous work has shown that timing of PC dendritic growth is different depending on maturation of specific lobules [29]. The formation of cerebellar lobules is under strong genetic control, as confirmed by the absence of lobulation in CXCR4-deficient mice [30], where granule cell precursors (gcps) fail to migrate properly to the internal granule cell layer, leading to a disorganized and irregular arrangement of PC dendritic trees. Distinct functional roles of different cerebellar lobules have been demonstrated by recent work that identified different firing patterns between lobules III-V and lobule X [31]. In addition, human subject fMRI studies point specifically to lobule VIII as being involved in timing of complex motor movement [32, 33].

In order to elucidate functions of neighboring PCs in different topological units, we used sagittal cerebellar sections near the midline to acquire confocal image stacks of GFP-expressing PC dendritic arbors in the sulcus and apex of the posterior cerebellum (Figure 1(a)). For anatomical consistency, we imaged PC arbors from the sulcus between lobules VIII and IX and the apex of lobule VIII in both young and old mice (Figures 1(a)–1(e)).

The contour tracing method extracted geometric information about PC dendritic arbor shape (Figure 1(g)) by imaging PC dendrites with high-resolution confocal microscopy to resolve distances (*μ*m) between neighboring dendrites (Figure 1(f)). Determination of dendritic arbor morphology enables us to understand the remarkably variable geometry of neuronal shape and to better understand how neighboring neurons gather information together.

### 3.2. PC Arbors in the Young Sulcus Exhibit Profound Morphological Differences Compared to Arbors in the Young Apex

To quantify geometric arbor shape differences between PC dendrites of sulcus and apex, we analyzed the number of primary dendrites emanating from each soma. PC arbors with two primary dendrites give rise to strikingly different geometric morphologies compared to arbors with only one. Dendritic arbors with only one primary dendrite are the typically portrayed PC shapes with a branching structure sitting atop a stalk-like main process (Figures 2(b) and 2(d)). Conversely, dendritic arbors with two primary dendrites diverge from each other within the sagittal plane, with one side of the arbor often more ramified than the other (Figures 2(a) and 2(c)). Dendritic arbors with two main primary dendrites usually populate the sulcus region of young and old mice (Figures 2(a) and 2(c)). These morphological differences are especially obvious in young mice when dendritic arbors are less well developed (Figure 2, panels (a) and (b) versus panels (c) and (d)).

In young mice, 72% of dendritic arbors in the sulcus have two primary dendrites, whereas in the apex, only 28% do (Figure 2(f)). This morphological difference is statistically significant in young mice (*P* < 0.05, *t*-test), while less pronounced in old mice, where 55% of the sulcus PC arbor population has two primary dendrites (Figures 2(f) and 2(h); *n* = 88, *P* = 0.6). While dendrites were not traced in the cerebellar bank in this study, PCs with two primary dendrites are less abundant in the bank than in the sulcus. Profound geometric differences of PC arbors in the sulcus imply a differential dendritic architecture of neighboring PCs dependent on anatomical location.

Sulcus PC arbors are also more frequently innervated by multiple climbing fibers relative to PC arbors of the apex and bank, further underscoring the uniqueness of the sulcus [4]. In addition, Sudarov and Joyner demonstrated that sulcus formation is associated with a peculiar aggregation of migrating gcps [5]. This aggregation, unique to the sulcus, is known as an anchoring center and seems to initiate invagination of the folia, giving rise to a fissure, which later becomes the concave sulcus. This process takes place as gcps migrate toward the internal granule cell layer, leaving their axons behind. The axons then bifurcate to become the future parallel fibers, synapsing with dendritic arbors. Thus, from a cytoarchitectural point of view, the peculiar gcps aggregation in the sulcus may influence the developing PC dendritic architecture. Future anatomical connectivity and functional studies are needed to determine whether neighboring sets of sulcus PCs and their afferents perform different downstream functions relative to their apex and bank counterparts.

### 3.3. Young Neighboring Sulcus PC Arbors Exhibit More Dendritic Field Overlap

How does the geometry of a sulcus PC arbor with two primary dendrites spreading along the sagittal plane influence neighboring dendritic arbors? We hypothesized that this shape might increase dendritic field overlap. Such overlap has functional implications because individual mouse PC dendrites receive approximately 100,000 excitatory inputs from unmyelinated parallel fibers [4]. These glutamatergic inputs are axons that run roughly perpendicular to the PC arbors (Figure 3(a)). Parallel fibers are roughly 1.5–3 millimeters long [34] and may contact a few hundred PC arbors, forming en passant synapses with neighboring PCs. The dendritic field shared by two neighboring PCs is likely to receive the same parallel fiber input (Figure 3(b)).

We first identified pairs of neighboring PCs that shared a common dendritic field. Next, we calculated the extent of this shared area in order to understand whether neighboring PC arbor geometries of sulcus and apex might give rise to different overall dendritic architectures, implying functional differences. Contour reconstructions permitted accurate representation of PC arbor geometries and were processed with an original algorithm that detected dendritic colocalization (space between branches was not analyzed). The algorithm performed the analysis in pairwise fashion and yielded the shared dendritic field as a surface area index (Figure 3(b)). This index was normalized to eliminate variable arbor size as a confounding factor and was used to compare the developing apex and sulcus (Figure 3(c)). The contour reconstruction method was suitable and efficient for the purpose of this study.

Neighboring PC arbors in young sulcus display greater dendritic field overlap than their counterparts in the apex (Figure 3(c); *n* = 88, *P* < 0.05, *t*-test). In contrast, in old mice, the shared dendritic field area between neighboring arbors was not significantly different between sulcus and apex (*n* = 88, *P* = 0.09). These results suggest a location-dependent developmental switch and dendritic developmental plasticity, where the ontogenetic effect on neighboring arbors potentially sharing similar afferent input depends on the dendritic architecture (Figure 3(c)).

It is well accepted that while single parallel fibers have a weak effect on the postsynaptic firing probability of PCs, the cumulative input of many fibers is the driving force responsible for PC activity [35, 36]. In the context of neighboring PCs, shared dendritic fields are very likely innervated by the same parallel fiber bundle, potentially promoting synchronous activity. Depending on the extent of dendritic field sharing, afferent input may regulate specific sets of PCs differently. In turn, PCs provide information to targeted cerebellar nuclei [37, 38], which are thought to regulate learned cerebellar movement behaviors, especially during development. Future experiments will attempt to determine whether sulcus PCs project to cerebellar nuclei with different connectivity patterns than those of the apex.

### 3.4. PC Arbor Geometry Is Altered by the Spatially Constricted Sulcus

To identify possible additional PC morphological characteristics specific to the sulcus, we analyzed PC arbor surface area and width in sulcus and apex. Contoured traces, tightly drawn around dendritic trees, enabled us to compute arbor surface areas. Somata were excluded from these analyses (Figure 4(a)). Mean dendritic arbor surface areas of young sulcus (mean 2272 *μ*m^2^ ± 840; range 912–3761 *μ*m^2^) and young apex (2334 *μ*m^2^ ± 888; 1050–4142 *μ*m^2^) did not differ significantly, and this insignificance persisted in old mice (sulcus, 3905 *μ*m^2^ ± 1680, 1070–6578 *μ*m^2^; apex, 4232 *μ*m^2^ ± 1773, 1484–7324 *μ*m^2^) (Figure 4(b)).

Next, we calculated the lateral span of PC arbors (Figure 4(c)) in order to explain the observed increase of shared dendritic field between neighboring young PCs. The upper portion of the arbors was measured from the left-most to the right-most branch tip (Figure 4(c)). Average arbor widths, like surface areas, did not differ significantly between sulcus and apex PCs in either young or old animals (young sulcus, 124 *μ*m ± 18; young apex, 117 *μ*m ±28, old sulcus, 137 *μ*m ±31, and old apex, 141 *μ*m ±45). As expected, during maturation, both surface area and widths of older dendritic arbors are significantly larger than those of young mice (Figures 4(b) and 4(d), *surface areas*, *n* = 88, *P* < 0.001; *widths*, *P* < 0.05, *t*-test). Dendritic tree width for old relative to young sulcus arbors only approached statistical significance (Figure 4(b), *n* = 88, *P* = 0.08). The small lateral growth is not surprising because PCs arbors grow first wider and then taller [39].

Population morphometric means obscure strikingly different individual PC arbor geometries (Figures 2 and 4). Previous reports on population means have asserted geometric uniformity in the cerebellar cortex [2, 40], while individual neuron level work points to varying dendritic morphologies playing a functional role in the propagation of action potentials [41], enrichment of computational power [42], or correlation with electrophysiological development [43].

Notably, both apex arbor surface areas and widths often exceeded those of sulcus arbors (Figures 4(b) and 4(d), open circles). This suggests that apex arbors are less spatially restricted, enabling more extensive ramification in the sagittal plane relative to sulcus arbors. The greater sulcus dendritic field overlap derives not from broader arbors, but from relative positions of extremely variable individual arbors, factors that are essential in order to understand functional connectivity between parallel fibers and PCs.

### 3.5. Developmental Implications on Dendritic Crowding in the Sulcus

Why are the most profound geometric differences of sulcus and apex PC arbors more prominent in young animals? How can the high frequency (72%) of young PC arbors with two primary dendrites be explained? What implication might these shapes have on shared dendritic overlap among young neighboring arbors? First, relative to the sulcus, the apex is less spatially restrictive to dendritic arbor growth. Secondly, P12 constitutes a developmental time window of active synaptogenesis [44], where the external granule cell layer is still present near the pial surface, and granule cells have not finished migrating through the molecular layer to make synaptic connections with postsynaptic PC dendrites [45]. At this stage, young dendritic arbors are in a stage of rapid growth, which may permit them to probe the environment more freely, invading the space of neighboring arbors within the spatial constraints of sulcus and apex. The combination of limited space and rapid dendritic growth may explain the significantly greater shared dendritic fields of young sulcus arbors. Conversely, PC arbors in old mice have completed their growth and although they experience more crowding in the sulcus, they exhibit similar amounts of shared dendritic field when compared to apex dendritic arbors (Figure 3(c), *P* = 0.1). A plausible explanation for this is that a compensatory structural change occurs in sulcus PC arbors.

### 3.6. Adult Sulcus Arbors Demonstrate Increased Intracellular Branch Crossings

To better understand the effect of spatial constraints on sulcus dendritic arbor development, we designed an algorithm to detect branch crossings of individual PC arbors (Figure 5(c)). There is a statistically significant increase in the number of intracellular branch crossings in old sulcus PC arbors relative to those of young sulcus (Figure 5(d), *n* = 82 total PCs, *P* < 0.05, Mann-Whitney *U* test). Adult sulcus PC arbors have more dendritic branch crossings than adult apex PCs (*P* < 0.05, Mann-Whitney *U* test). Differences in the number of intracellular branch crossings between sulcus and apex are less pronounced in young mice; however, their arbors are immature. These results imply that adult sulcus arbors may compensate for the space limitations of the sulcus by developing a compact dendritic arbor within the sagittal plane.

Previous work concluded that PC dendritic branch crossings provide evidence for dendritic arbor multiplanarity [6], retained into adulthood. We observed that intracellular branch crossings almost always occurred between small, distal dendrites ~0.5–1.75 *μ*m in diameter. However, in 3D space, crossed branches of the same arbor did not contact each other, and, as could be readily visualized by rotating the PC, the small interbranch distances usually did not extend beyond the plane of the main dendritic trunk. Primary dendrites reportedly range from 4 to 6 *μ*m in thickness [11, 17] and from 8 to 9 *μ*m, including the 2–4 *μ*m-space between neighbors [46]. Our measurements of primary dendrite thickness agree well with those cited above (results not shown). In addition, we measured the thickness of the entire PC arbor (its depth in the mediolateral plane) (Figures 5(a) and 5(b)). Sulcus trees tend toward greater thickness than those of apex (Figure 5(b)). Some sulcus arbors attain nearly 40 *μ*m thickness, suggesting that they may be multiplanar. This is most likely due to unusually large branches in the mediolateral plane, rather than to smaller branch crossings. These results suggest that space limitations within the sagittal plane of the sulcus cause dendritic branches to expand into the mediolateral plane. Because our study focused mainly on the sagittal dimension, we did not intend initially to explore the planarity of PC trees. Dendritic planarity would be best addressed with coronally cut slices or slices transverse to the length of the lobule.

Some studies have reported repulsion between neighboring dendrites [47]. Intracellular branch crossings and shared dendritic fields are structural attributes that occur without interdendrite contact; thus, it seems likely that PC dendrites do not experience strong steric hindrances in intact brain.

## 4. Conclusions

Due to its organized anatomy and well-studied circuitry, the cerebellum is an ideal site for drawing functional inferences from neuronal geometry. By combining thin optical slicing on a fast scanning confocal microscope with an adapted software tool to trace fluorescent dendrites, we demonstrated that spatial constraints of the sulcus alter PC dendritic arborization relative to that of the apex. First, the majority of young sulcus arbors have two primary dendrites that diverge in the sagittal plane. Second, young neighboring sulcus arbors overlap to a greater degree. Third, adult sulcus dendrites manifest more intraarbor branch crossings and tend toward narrower arbors, resulting in more compact structure sagittally. Finally, this morphological plasticity is ontogenetic.
